# *CREBBP* is a target of epigenetic, but not genetic, modification in juvenile myelomonocytic leukemia

**DOI:** 10.1186/s13148-016-0216-3

**Published:** 2016-05-05

**Authors:** Silvia Fluhr, Melanie Boerries, Hauke Busch, Aikaterini Symeonidi, Tania Witte, Daniel B Lipka, Oliver Mücke, Peter Nöllke, Christopher Felix Krombholz, Charlotte M Niemeyer, Christoph Plass, Christian Flotho

**Affiliations:** Division of Pediatric Hematology and Oncology, Department of Pediatrics and Adolescent Medicine, University Medical Center, Mathildenstrasse 1, 79106 Freiburg, Germany; Hermann Staudinger Graduate School, University of Freiburg, Freiburg, Germany; Institute of Molecular Medicine and Cell Research, University of Freiburg, Freiburg, Germany; German Cancer Research Center (DKFZ), Heidelberg, Germany; German Cancer Consortium (DKTK), Heidelberg, Germany; Division of Epigenomics and Cancer Risk Factors, German Cancer Research Center, Heidelberg, Germany

**Keywords:** Juvenile myelomonocytic leukemia, DNA methylation, Epigenetics, *CREBBP*

## Abstract

**Background:**

Juvenile myelomonocytic leukemia (JMML) is a myeloproliferative neoplasm of childhood whose clinical heterogeneity is only poorly represented by gene sequence alterations. It was previously shown that aberrant DNA methylation of distinct target genes defines a more aggressive variant of JMML, but only few significant targets are known so far. To get a broader picture of disturbed CpG methylation patterns in JMML, we carried out a methylation screen of 34 candidate genes in 45 patients using quantitative mass spectrometry.

**Findings:**

Five of 34 candidate genes analyzed showed recurrent hypermethylation in JMML. cAMP-responsive element-binding protein-binding protein (*CREBBP*) was the most frequent target of epigenetic modification (77 % of cases). However, no pathogenic mutations of *CREBBP* were identified in a genetic analysis of 64 patients. *CREBBP* hypermethylation correlated with clinical parameters known to predict poor outcome.

**Conclusions:**

This study supports the relevance of epigenetic aberrations in JMML pathophysiology. Our data confirm that DNA hypermethylation in JMML is highly target-specific and associated with higher-risk features. These findings encourage the development of prognostic markers based on epigenetic alterations, which will be helpful in the difficult clinical management of this heterogeneous disease.

**Electronic supplementary material:**

The online version of this article (doi:10.1186/s13148-016-0216-3) contains supplementary material, which is available to authorized users.

## Findings and conclusions

Risk assessment in juvenile myelomonocytic leukemia (JMML) is important for clinical decision-making as this aggressive myeloproliferative neoplasm is generally unresponsive to conventional chemotherapy and most (but not all) patients need early allotransplantation [1, 2]. Several clinical parameters have been well-established to predict poor outcome [2], but the picture is less clear at the molecular level. Specifically, if and how the set of genetic mutations in leukemic cells contributes to clinical risk remains a matter of ongoing debate [2–4]. Recent research on epigenetic dysregulation in JMML has provided a growing body of evidence that high-risk cases of JMML are characterized by specific CpG island hypermethylation which integrates the clinical and genetic risk factors identified so far [5–7]. To provide a broader picture of epimutations in JMML, we carried out a DNA methylation analysis of 34 candidate genes in leukemic cells from 45 children with JMML.

## Aberrant methylation of *CREBBP*, *MPO*, *SLC12A8*, *HIC1*, and *TCF4* in JMML

**Fig. 1:**
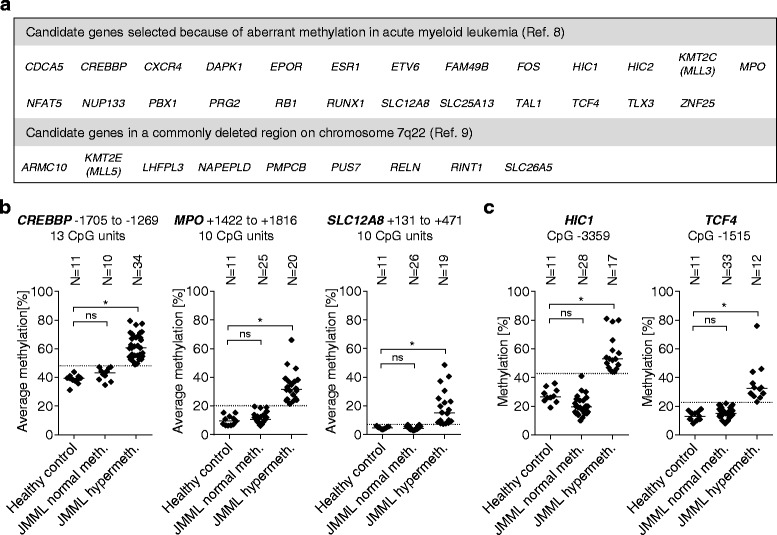
DNA methylation analysis of 34 genes in 45 children with JMML and 11 healthy controls. **a** Candidate genes for analysis of CpG island methylation in JMML. **b** *CREBBP*, *MPO*, and *SLC12A8* were recurrently hypermethylated in JMML. CpG methylation of the indicated target regions was determined in granulocyte DNA from children with JMML and healthy control subjects using quantitative mass spectrometry. The average level of methylation across all CpG units is shown for each sample. A JMML sample was categorized as hypermethylated if the methylation level exceeded three standard deviations above the mean observed in healthy controls. The upper limit of normal methylation is illustrated by a dotted line. The significance of differences was calculated using the Mann-Whitney test and is indicated as follows: *p ≤ 0.0014 (Bonferroni threshold for significance level *p* ≤ 0.05 after correction for 36 tests, i.e., 34 genes plus *HIC1* CpG 3359 and *TCF4* CpG 1515); ns, *p* > 0.0014. **c** The *HIC1* and *TCF4* genes had normal methylation in JMML when averaging all CpG units in the region of analysis, but each region contained a single CpG unit with significant hypermethylation in JMML

We assessed the CpG island methylation of 34 candidate genes (Fig. 1a) in granulocyte DNA from 45 children with JMML and 11 healthy adult individuals using quantitative high-resolution mass spectrometry. Selection of twenty-five candidate genes followed a methylation panel in acute myeloid leukemia published previously [8]. Considering that monosomy 7 is the most frequent cytogenetic abnormality observed in JMML cells [2], and reasoning that a two-hit mechanism of tumor suppressor inactivation might involve hypermethylation of the second allele after deletion of one allele, we selected an additional nine genes because of their location in a ~2.5 mega base pair region on chromosome segment 7q22 which is commonly deleted in myeloid malignancies [9]. In the cohort used here, monosomy 7 was present in 11/45 cases (24 %). Detailed information on the DNA regions interrogated by the mass spectrometry assays is given in Additional file 1: Table S1.

We defined a gene as hypermethylated in a JMML sample if the average CpG methylation in the region of analysis exceeded three standard deviations above the mean observed in granulocytes of 11 healthy control subjects. Using this definition, 34 of 44 JMML cases (77 %) showed hypermethylation of the cAMP-responsive element-binding protein-binding protein (*CREBBP*) gene (the assay was uninformative in one case), 20 of 45 (44 %) were hypermethylated in the *MPO* gene, and 19 of 45 (42 %) carried a hypermethylated *SLC12A8* gene (Fig. 1b). Most CpG units analyzed in the *HIC1* and *TCF4* genes had normal methylation in JMML, but each region contained a single CpG unit near its border with significant hypermethylation in JMML (Fig. 1c). This observation may hint at aberrant methylation in neighboring regions not covered by the assay. A group-wise comparison of methylation levels using Mann-Whitney test with Bonferroni correction showed for each gene a highly significant difference between the “hypermethylated JMML” group and healthy controls but no difference between “normal methylation JMML” and controls (Fig. 1b, c). The DNA methylation level of the remaining 29 genes was similar between JMML samples and healthy granulocytes or the difference was so small that no biological significance would be expected (Additional file 1: Table S1).

The analysis demonstrates that CpG hypermethylation in JMML is highly locus-specific. Although a significant proportion of JMML cases are characterized by aberrant DNA methylation, the hypermethylation events are confined to a limited array of genetic regions. This observation fits well with three previous studies where 11 of 15 candidate regions did not show any aberrant methylation in 127 cases analyzed [5], or where hypermethylation was restricted to only one of two CpG islands of the *RASA4* locus [6] or one of three CpG islands of the *AKAP12* locus [10]. Contrary to the two-hit hypothesis formulated above, none of the nine 7q22 genes exhibited frequent hypermethylation in JMML, whether monosomy 7 was present or not.

## High-resolution DNA methylation analysis of the *CREBBP* CpG island in JMML

**Fig. 2:**
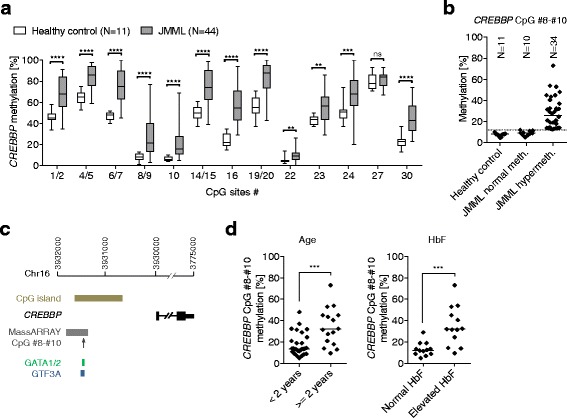
Epigenetic dysregulation of *CREBBP* in JMML and association with clinical parameters indicating poor outcome. **a** The high-resolution DNA methylation profile of a CpG-rich 437-base pair region upstream of the *CREBBP* transcription start site revealed high methylation variation between CpG units under physiological conditions and frequent hypermethylation in JMML. The *box plots* depict the methylation level of 13 *CREBBP* CpG units in granulocyte DNA from 11 healthy control subjects (*open boxes*) and 44 patients with JMML (*filled boxes*). The band inside the box represents the median, and the bottom and top of the box represent the first and third quartiles. The ends of the whiskers correspond to the minimum and maximum values. The significance of differences was calculated using the Mann-Whitney test and is indicated as follows: ns, *p* > 0.05; **p ≤ 0.01; ***p ≤ 0.001; ****p ≤ 0.0001. **b** Methylation of *CREBBP* CpG sites #8 to #10 was low under physiological conditions but increased dramatically in most JMML cases. The average level of methylation across CpG units #8–#10 is shown for each sample. A JMML sample was categorized as hypermethylated if the methylation level exceeded three standard deviations above the mean observed in healthy controls. The upper limit of normal methylation is illustrated by a *dotted line*. **c** In silico prediction of transcription factor binding sites (TFBIND) revealed binding motifs for GATA1/2 and GTF3A at CpG sites #8 to #10. **d** *CREBBP* CpG #8–#10 methylation was associated with older age (≥2 years) and elevated fetal hemoglobin (HbF) at diagnosis, two established predictors of poor outcome. ***p ≤ 0.001 (Mann-Whitney test)

The *CREBBP* gene was hypermethylated in 77 % of JMML cases. CREBBP is a histone acetyltransferase and functions as transcriptional co-activator of a large number of regulatory proteins [11]. Mice with a null mutation in one *CREBBP* allele develop a myelodysplastic/myeloproliferative neoplasm [12]. Decreased expression of CREBBP facilitates Ras-induced transformation [13]. The *CREBBP* methylation assay used here covered 13 CpG units in a 437-base pair region located 1.5 kilobases upstream of the transcription start site. Most *CREBBP* CpG units were characterized by remarkable baseline methylation in healthy controls which increased to even higher levels in JMML (Fig. 2a). However, three *CREBBP* CpG units (containing CpG sites #8, #9, #10, and #22) were virtually unmethylated in healthy granulocytes. Of these, CpG sites #8 to #10 acquired particularly high hypermethylation in JMML (Fig. 2b), raising the possibility of specific functional importance. In silico analysis using TFBIND [14] revealed that CpG sites #8 to #10 may interfere with binding motifs for the transcription factors GATA1/2 and GTF3A (Fig. 2c).

## Somatic *CREBBP* mutations are uncommon in JMML

We next studied if the *CREBBP* locus was not only subject to epigenetic modification in JMML, but also to genetic mutation. We analyzed leukemic granulocyte DNA from 64 children with JMML (17 of which overlapped with the 44-patient series used for epigenetic analysis) for *CREBBP* exon sequence variants using the Agilent SureSelect v4.0 capture technique and deep-sequencing, resulting in an average *CREBBP* exon depth of 82 reads. Eighty percent of *CREBBP* exons were covered by at least 50 reads. No insertions or deletions in the *CREBBP* gene were found in any JMML sample. After removing common non-pathogenic single-nucleotide polymorphisms documented in public databases such as dbSNP, we identified 10 non-synonymous sequence variants in 11 cases of JMML (Additional file 1: Table S2). All except one (c.A493G) were listed in at least one of four exome variation resources (Exome Variant Server, ExAC browser, 1000 Genomes, dbSNP) with minor allele frequencies of less than 0.1 %. Three variants (c.A5933G, c.A2728G, c.G2941A) corresponded to alterations associated with Rubinstein-Taybi syndrome [15, 16]. However, indicative clinical features (facial dysmorphism, thumb or toe malformation, intellectual disability) were documented in none of the 11 children. Importantly, we did not detect any of the *CREBBP* histone acetyltransferase domain mutations described as pathogenic somatic events in lymphoblastic leukemia or lymphoma [17–20]. DNA from paired non-hematopoietic tissue was available in 5 of 11 JMML cases with non-synonymous *CREBBP* sequence variants, enabling us to test the germline status. In all cases, the variants were present in non-leukemic tissue. The pathogenic significance of these germline *CREBBP* variants in JMML patients without syndromic features remains unclear. Taken together, the data indicate that the coding sequence of *CREBBP* is not a relevant target of somatic alteration in JMML, in agreement with the absence of *CREBBP* mutations from myeloproliferative neoplasms in adults [21].

## *CREBBP* methylation is associated with established hematologic and clinical parameters indicating an aggressive JMML phenotype

It was repeatedly shown that DNA hypermethylation at specific target genes in JMML is associated with clinical parameters indicating poor prognosis [5–7, 10]. To test if this relationship was also true for *CREBBP* methylation, we focused on CpG sites #8 to #10 because these sites were unmethylated in healthy individuals but had high methylation variation in JMML. The level of methylation at these sites was significantly associated with older age and elevated fetal hemoglobin at diagnosis (Fig. 2d and Additional file 1: Table S3), two main predictors of reduced survival [1, 2]. In addition, methylation at CpG sites #8 to #10 was associated with the category of Ras pathway mutation (Additional file 1: Table S3), being considerably higher in JMML cases with *PTPN11* mutation or neurofibromatosis type 1 (NF1) than in JMML with *KRAS*, *NRAS*, or *CBL* mutation. The relevance of the mutation category to disease course and outcome of JMML remains controversial [2–4, 22], but higher *CREBBP* methylation in JMML with *PTPN11* mutation or NF1 is consistent with the prevailing opinion that these subgroups are more aggressive. Variable correlation with age, high fetal hemoglobin, mutation, or karyotype but no other parameters was also found for *MPO*, *SLC12A8*, and *HIC1* CpG 3359. The small group size and heterogeneous treatment precluded meaningful outcome analyses. However, we noted that five events of death occurred in the *CREBBP* hypermethylation group (5/34 patients, 15 %) compared to no such event in the group with normal *CREBBP* methylation (0/10 patients); the median follow-up time was 6.0 years for all 44 patients. In summary, the data strengthen the concept that higher-risk cases of JMML carry a distinct DNA methylation phenotype. Future studies are needed to elucidate the functional role of coordinated DNA hypermethylation at specific loci in the JMML-initiating cell.

## Study design

### Patient samples

We used clinical material from German JMML patients registered in the European Working Group of MDS in Childhood (EWOG-MDS) studies “98” and “2006” after obtaining informed consent and ethics approval (University of Freiburg, reference number EK247/05). Patients with Noonan syndrome (*PTPN11* or *KRAS* germline mutation) were excluded. Clinical characteristics are given in Additional file 1: Table S3.

## High-resolution quantitative DNA methylation analysis

Leukemic granulocytes from 45 patients (bone marrow, *N* = 36; peripheral blood, *N* = 9) were enriched by Ficoll centrifugation (Biochrom). Genomic DNA was isolated using the Gentra Puregene kit (Qiagen) and bisulfite converted using the EZ DNA methylation kit (Zymo Research). The MassARRAY EpiTYPER assay (Agena Bioscience) was used to assess CpG island methylation at 34 target regions (Fig. 1a and Additional file 1: Table S1). Primer sequences are listed in Additional file 1: Table S4.

## *CREBBP* sequence analysis

Library construction and exon region capture of fragmented genomic DNA isolated from leukemic granulocytes of 64 patients were done using TruSeq (Illumina) and SureSelect v4.0 (Agilent). 100-base pair paired-end sequencing was performed on HiSeq2000 instruments (Illumina). The reads were aligned to the hg19 reference genome. *CREBBP* single-nucleotide polymorphisms and indels were identified according to standard recommendations (Genome Analysis Toolkit, Broad Institute).

## Additional file

Additional file 1: This file contains additional details on methods and Tables S1–S4. (PDF 218 kb)

## Abbreviations

JMML: juvenile myelomonocytic leukemia
CREBBP: cAMP-responsive element-binding protein-binding protein
NF1: neurofibromatosis type 1
