# Supplementary material for: CREBBP is a target of epigenetic, but not genetic, modification in juvenile myelomonocytic leukemia
Source: Clin Epigenetics. 2016 May 5;8:50. doi: 10.1186/s13148-016-0216-3 (PMC4858931; doi:10.1186/s13148-016-0216-3)
Supplement: Additional file 1: — This file contains additional details on methods and Tables S1–S4. (PDF 218 kb) [file 13148_2016_216_MOESM1_ESM.pdf]

Table S1. DNA methylation of 34 candidate regions in JMML and healthy controls.

Gene	Gene selection criteria	Region of analysis, position relative to transcription start site	Number of CpG units analyzed	Methylation range, healthy controls [%]	Threshold hypermethylation [%]*	Methylation range, JMML [%]	Hypermethylated JMML cases
CREBBP	Ref. 8	-1705 to -1269 (variant 1)[#]	13	31.3 – 43.8	48.2	34.7 – 79.5	34 / 44 (77%)
MPO	Ref. 8	+1422 to +1816	10	6.2 – 15.3	20.2	6.0 – 66.0	20 / 45 (44%)
SLC12A8	Ref. 8	+131 to +471 (variant 1)[#]	10	3.4 – 6.1	7.1	2.8 – 48.5	19 / 45 (42%)
HIC2	Ref. 8	-821 to -389	15	5.1 – 7.7	8.6	3.8 – 15.6	13 / 44 (30%)
TLX3	Ref. 8	-71 to 324	13	4.8 – 7.3	8.3	3.8 – 21.4	11 / 45 (24%)
TAL1	Ref. 8	+887 to +1360 (variant 1)[#]	10	4.3 – 5.3	5.8	3.7 – 7.7	8 / 44 (18%)
RINT1	7q22	-90 to +328	11	3.1 – 4.1	4.3	2.7 – 6.7	8 / 45 (18%)
TCF4	Ref. 8	-1540 to -1171 (variant 1)[#]	11	2.9 – 4.9	5.7	2.5 – 16.6	8 / 45 (18%)
LHFPL3	7q22	-28 to +403	9	2.1 – 4.0	4.4	2.2 – 8.4	8 / 45 (18%)
SLC26A5	7q22	+468 to +760 (variant a)[#]	8	3.5 – 6.4	8.1	2.0 – 15.6	7 / 45 (16%)
ESR1	Ref. 8	+224 to +634 (variant 1)[#]	9	3.6 – 5.8	6.8	3.9 – 41.7	6 / 45 (13%)
HIC1	Ref. 8	-3558 to -3235 (variant 1)[#]	4	8.5 – 18.8	22.2	6.0 – 28.8	4 / 45 (9%)
ARMC10	7q22	-163 to +228 (variant a)[#]	9	1.3 – 2.1	2.5	0.9 – 2.7	3 / 42 (7%)
NAPEPLD	7q22	-23 to +290 (variant 1)[#]	7	0.7 – 2.1	2.8	0.7 – 7.6	3 / 45 (7%)
KMT2E (MLL5)	7q22	-942 to -514 (variant 1)[#]	10	1.1 – 2.5	3.1	1.3 – 3.5	2 / 44 (5%)
FAM49B	Ref. 8	-651 to -233 (variant 1)[#]	6	1.5 – 15.0	15.1	1.5 – 15.5	2 / 44 (5%)
PUS7	7q22	+249 to +662 (variant 2/3)[#]	10	3.2 – 6.1	6.9	2.8 – 9.7	2 / 44 (5%)

EPOR	Ref. 8	+539 to +945 (variant 1)#	12	2.0 – 4.8	5.3	1.6 – 7.5	2 / 45 (4%)
CXCR4	Ref. 8	-1561 to -1229 (variant 1)#	9	2.2 – 3.4	3.7	1.9 – 4.4	2 / 45 (4%)
ETV6	Ref. 8	-964 to -695	14	2.5 – 4.2	4.9	2.3 – 8.4	2 / 45 (4%)
NFAT5	Ref. 8	+447 to +867 (variant 1)#	12	1.7 – 3.6	4.2	1.2 – 6.6	2 / 45 (4%)
PMPCB	7q22	-52 to +998	6	1.0 – 3.0	3.8	1.2 – 4.0	1 / 45 (2%)
NUP133	Ref. 8	-280 to +103	11	1.1 – 2.9	3.3	1.3 – 3.7	1 / 45 (2%)
RB1	Ref. 8	+247 to +481	8	1.1 – 2.1	2.5	1.1 – 2.6	1 / 45 (2%)
ZNF25	Ref. 8	-373 to -64	9	1.8 – 3.3	3.7	1.2 – 4.1	1 / 45 (2%)
RUNX1	Ref. 8	-385 to +10 (variant 2/3)#	11	1.8 – 3.0	3.5	1.7 – 3.5	0 / 45 (0%)
DAPK1	Ref. 8	-107 to +136 (variant 1)#	6	2.5 – 4.0	4.6	2.0 – 3.5	0 / 45 (0%)
SLC25A13	Ref. 8	-19 to +368 (variant 1)#	9	2.1 – 4.1	5.4	1.8 – 4.4	0 / 45 (0%)
PRG2	Ref. 8	-36546 to -36169 (variant 1)#	12	1.0 – 2.3	2.3	0.8 – 2.2	0 / 45 (0%)
FOS	Ref. 8	-1535 to -1210	13	2.2 – 4.0	4.6	2.5 – 4.5	0 / 45 (0%)
RELN	7q22	-845 to -458 (variant 1)#	8	1.1 – 3.4	3.9	0.9 – 3.0	0 / 45 (0%)
KMT2C (MLL3)	Ref. 8	-28349 to -28061	10	2.5 – 4.9	5.7	2.4 – 5.7	0 / 45 (0%)
CDCA5	Ref. 8	-154 to +290	12	2.6 – 4.9	5.6	2.6 – 5.1	0 / 45 (0%)
PBX1	Ref. 8	+16763 to +17233 (variant 1)#	16	86.3 – 93.1	96.4	40.3 – 94.6	0 / 45 (0%)

* Three standard deviations above the mean of 11 healthy controls

Transcript variants according to NCBI RefSeq Gene, GRCh37/hg19

Table S2. *CREBBP* sequence variants in 64 cases of JMML.

Patient ID	Ras pathway category	*CREBBP* NM_004380	*CREBBP* NM_001079846	Variant allele frequency in this sample	Germline	Estimated population frequency			dbSNP138
						Exome Variant Server	ExAc Browser	1000 Genomes	
D124	PTPN11	exon16:c.C3238G:p.P1080A	exon15:c.C3124G:p.P1042A	0.41	Unknown	0.0001	-	-	rs373586649
D448	Quadruple-negative	exon19:c.A3611T:p.Y1204F	exon18:c.A3497T:p.Y1166F	0.45	Unknown	0.0001	0.0001	-	rs200346970
D561	NF1	exon31:c.A5933G:p.N1978S	exon30:c.A5819G:p.N1940S	0.45	Unknown	0.0043	0.0088	0.002	rs112906840
D567	PTPN11	exon14:c.A2728G:p.T910A	exon13:c.A2614G:p.T872A	0.49	Unknown	0.0023	0.0023	0.000	rs143247685
D598	KRAS	exon07:c.C1651A:p.L551I	exon6:c.C1537A:p.L513I	0.36	Unknown	0.0087	0.0100	0.006	rs61753381
D763	PTPN11	exon07:c.C1651A:p.L551I	exon6:c.C1537A:p.L513I	0.40	Yes	0.0087	0.0100	0.006	rs61753381
D766	PTPN11	exon15:c.G2941A:p.A981T	exon14:c.G2827A:p.A943T	0.34	Yes	0.0041	0.0039	0.002	rs61753380
D823	Quadruple-negative	exon02:c.A493G:p.S165G	exon2:c.A493G:p.S165G	0.59	Yes	-	-	-	-
D854	NRAS	exon31:c.C6449T:p.P2150L	exon30:c.C6335T:p.P2112L	0.43	Yes	-	<0.0001	-	-
D903	NF1	exon14:c.C2678T:p.S893L	exon13:c.C2564T:p.S855L	0.35	Yes	0.0014	0.0008	<0.001	rs142047649
D953	PTPN11	exon16:c.C3230T:p.P1077L	exon15:c.C3116T:p.P1039L	0.46	Unknown	-	0.0082	-	-

Table S3. Correlation of *CREBBP* CpG #8–#10 methylation with clinical and hematologic characteristics of JMML patients.

	N	Correlation with *CREBBP* methylation		Category	N	*CREBBP* methylation [%]	
		**Spearman**	**p**			**Median**	**p**
Total cohort	44				44	18.5	
**Age [years]**	44	0.471	<0.01				
				<2 years	28	13.8	
				>= 2 years	16	32.0	<0.01
**Sex**	44						
				Male	29	14	
				Female	15	29	n.s.
**Leukocytes [10^9/L]**	44	0.077	n.s.				
				<30	22	14.3	
				>=30	22	19.0	n.s.
**Platelets [10^9/L]**	43	0.074	n.s.				
				Transfused before diagnosis	1		
				<50	11	14.0	
				50-100	11	18.0	
				>=100	21	21.0	n.s.
**Hemoglobin [g/dL]**	41	-0.056	n.s.				
				Transfused before diagnosis	3		
				<10	27	18.0	
				>=10	14	21.0	n.s.
**Myeloblasts (PB) [%]**	44	0.220	n.s.				
				<2	20	14.0	
				>=2	24	24.3	n.s.
**Myeloblasts (BM) [%]**	43	0.180	n.s.				
				Missing	1		
				<5	16	17.3	
				>=5	27	19.0	n.s.
**Monocytes (PB) [%]**	44	0.110	n.s.				
				<10	6	21.3	
				10-19	15	18.0	
				>=20	23	19.0	n.s.
**Monocytes (BM) [%]**	43	0.226	n.s.				
				Missing	1		
				<5	14	16.5	
				>=5	29	19.0	n.s.
**Spleen size [cm below the costal margin]**	42	-0.115	n.s.				
				Missing	2		
				<5cm	27	19.0	
				>=5cm	15	14.5	n.s.
**Hemoglobin F (age-adjusted)**							
				Normal	12	12.3	
				Elevated	13	32.0	<0.01
				Missing	19		
**Karyotype**							
				Normal	26	14.0	
				Aberrant	15	27.5	n.s.
				Missing	3		
**Mutation**							
				NF1	5	32.5	
				PTPN11	15	29.0	
				KRAS	6	13.8	
				NRAS	5	13.5	
				CBL	8	9.3	<0.01
				No mutation	2		
				Missing	3		

Abbreviations: n.s., not significant; PB, peripheral blood; BM, bone marrow

Nonparametric statistics were used to test *CREBBP* methylation for differences between 2 subgroups (Mann-Whitney test) or more than 2 subgroups (Kruskal-Wallis test). P values below or equal to 0.05 were considered to be statistically significant.

Table S4. DNA methylation analysis using mass spectrometry (EpiTYPER, Agena Bioscience).

Target	Forward primer	Reverse primer
ARMC10	TTATTTGATTTGGATGTGGAGAAAG	TTCTCCTTAAAATAATTCTTCAACCTC
CDCA5	GGGAGGGAAGAGGTTATTTTTTAT	ACCACTCTCCCCAAAACTCCTAC
CREBBP	GATATTAGGGAGTGAGGGGGTT	AAAAAACAATCTCCCAAATAAAAAC
CXCR4	TTGTGTTGGGAGATTGGTATAGTTT	TACATATATCTCCCCCTTAAATCCC
DAPK1	AGTTTAGTAATGTGTTATAGGTG	ACCAATAAAAACCCTACAAAC
EPOR	GGTTGATTTGGTGTTAAGGTTTTT	CCCCTAATTCCCAAAAACAAA
ESR1	TTTTTTATATTAAAGTATTTGGGATGG	TTCTCCAAATAATAAAACACCTACTAACC
ETV6	AAGAGAATTTATTAGGAAATGGGAGA	TTTAATAACTACCCTACAACCTTCCC
FAM49B	GGTTGGGGTTTTTTAGTTTTTTAG	ACCCACTAAATATTTTTACCCAAAATC
FOS	GGAGGTAAGGTGTTTTAGAGTGTGT	TTCCCTATTACTATCTATAACAAAATCTCC
HIC1	GTTGTTTATTATTTTTGGGGAGGTT	CCCTCCTCAATTCCTAAACCTAA
HIC2	GTTTTTGGGAAGGTTATTTGTATGG	AATAAACTTACCCCCTTTTAACCCT
KMT2C (MLL3)	GGTTTTTTAGTGGTTAGGGAGAGAG	AAAACTAACAACCTATCAAATCCACTTT
KMT2E (MLL5)	GTTTATTAGGGGTTGAGGAGGTT	AAAAAACCCCCAAAACAAAAA
LHFPL3	GAGGATGTAGATTTTGAAATTGGTG	AACTACCCCTACAAATCAACTCCC
MPO	GTTGGGGGTGGTTGTAGGAAT	CAACTAACCCCATTACATAAACATAAA
NAPEPLD	TGTAATTTGGTAATTTGTAGGGAAGA	ATAACCTCTCTACTACCTCCTCCAC
NFAT5	AGATAGGGAGATAGGGAGATAGGGT	AAAAAATAATAACCCTACACTCAAAAC
NUP133	GGTTGGGAATATGATTTTAAGGAGT	TTCACAAAAAATTCAAAAACTACCA
PBX1	TTTTTGGAATTAAGAAATAGTGGAGAA	AAAATAATAAAAACACAACAAACCCC
PMPCB	TGGTTAAATATGTATTTTTTAGTAGTTGG	AAAATAAAACCCACCTCCCTACTC
PRG2	AGTGGGAGGTTTGTTTTAGTTTTTT	AACCATTCTACTACCAAAAATACCCC
PUS7	GGGTTGGATTTATAGAAGGTAGG	TAACCCTAACCCCAACCCCA
RB1	TTTTTTGAGGAGGATTTAGAGTAGG	CAAAATCCTATCACCATTCTACAAA
RELN	GTTTAGTTGTTTGAAGGGGAAGGT	ACAACACAAATCACCATTTCCAAAC
RINT1	TTTTAATAAAGTGGAGGGGATTTTTAT	ATACCACCTCAATAAACCACCAATA
RUNX1	TTGGTTTTATGAATGAGAGTGTTTG	ACCTACTTTCTTTTTCCAAATCTCC
SLC12A8	TAGTTAGGAGTTAGGTTTGGGGTTA	TAACAAATAAAAAACCAAAACACCC
SLC25A13	GTTAAGGTTGGGTTTAGTTAATGGG	AAAAATAACCCCCTCCCTCC
SLC26A5	AGAGTTTTTATGATGGTAGAGTATTTGT	CAATTCTACCTCTAACCCTCTTTCC
TAL1	GTTGGATTTTGTGTGGTTTTGTT	CCTAATAAATATACCCATTATCCTTTC
TCF4	GTTTATAAAGAGAAGGAGTT	ACCTAAAAATATCTCACTTC
TLX3	GGTTTAAGAAAGATGATATAGAGTTTGT	ACTCAAATTCACACTATAAAATCCC
ZNF25	AAAATTGTTTATTAGTGTTTTTTGGAG	AAATCCTCTCCCCACCCTAAA

Each target region was PCR amplified in such a way that a T7 promoter was introduced at the reverse strand for subsequent *in vitro* transcription of the reverse strand. The resulting RNA was subjected to uracil-specific cleavage which yielded small fragments (termed CpG units) containing one to several CpG sites. The CpG methylation status translated into

difference in fragment weight due to C to T conversion after bisulfite treatment. The fragments were quantified by MALDI-TOF mass spectrometry. CpG units with low quality data (bad performance of methylation standards, low signal intensity or lack of data for more than 50% of the analyzed samples) were excluded.
